# A rare case of chest wall lipoma growing into the pleural cavity: a case report

**DOI:** 10.1186/s13019-021-01576-x

**Published:** 2021-07-12

**Authors:** Hyo Joon Jang, Bu Hyeon Choi, Seong Oh. Park

**Affiliations:** 1 Department of Thoracic and Cardiovascular Surgery, Hanyang University College of Medicine, Seoul, South Korea; 2 Department of Plastic and Reconstructive Surgery, Hanyang University College of Medicine, Seoul, South Korea

**Keywords:** Lipoma, Chest wall lipoma, Deep-seated lipoma, Dumbbell shape

## Abstract

**Background:**

Several cases of lipoma in unusual locations in the thorax have been reported. Appropriate surgical treatment depending on the location and shape is often required.

**Case presentation:**

We herein report an extremely rare case of a chest wall lipoma growing into the pleural cavity. The tumor was successfully removed without damaging the capsule by a combination of direct and thoracoscopic approaches.

**Conclusions:**

Chest wall lipomas growing into pleural cavity can be successfully treated by a combination of direct and thoracoscopic approaches.

## Background

Lipomas are one of the most common soft tissue tumors and are typically located in the subcutaneous layer. Typical deep-seated lipomas are relatively rare and are located in the subfascial tissue (deep lipomas) or on bone surfaces (parosteal lipomas). In the thoracic area, several cases of lipomas in unusual locations such as intracardiac lipomas [1], pleural lipomas [2–4], and intramuscular lipomas [5, 6] have been reported. We herein report an extremely rare case of a chest wall lipoma penetrating the pleural cavity.

## Case presentation

A 60-year-old male patient visited the department of plastic and reconstructive surgery with a palpable mass in the right subscapular area. The mass was about 6 cm in size, round and rubbery, and was assumed to be located in the deep layer. An intramuscular or a submuscular lipoma was suspected.

**Fig. 1:**
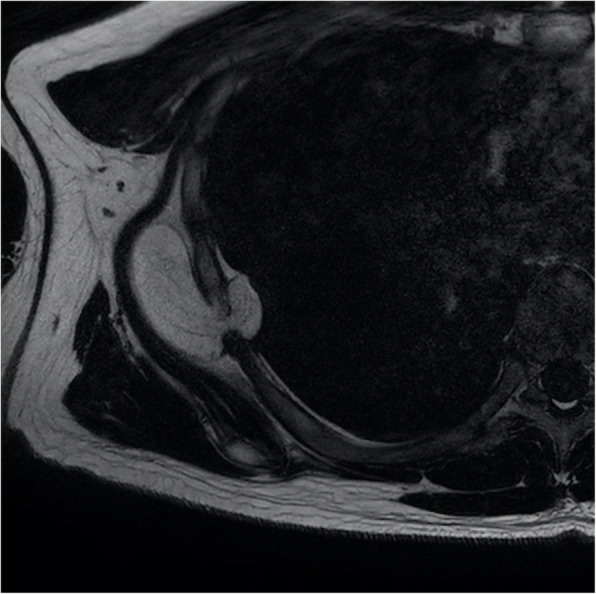
MR image revealing an encapsulated fatty mass. The mass was located between the right serratus anterior muscle and the right rib cage. The size of the mass was estimated to be 5.5 × 2.3 × 5.2 cm. The mass was insulated in the right 6th–7th intercostal space resulting in herniation into the pleural cavity

Magnetic resonance (MR) image showed an encapsulated fatty mass between the right serratus anterior muscle and the right rib cage. The size of the mass was estimated to be 5.5 × 2.3 × 5.2 cm. The mass was insulated in the right 6th–7th intercostal space resulting in herniation into the pleural cavity. Thin internal septations were also observed (Fig. 1). The assessment from MR imaging was that it was either an intermuscular lipoma or a well-differentiated liposarcoma.

It was thought that both direct and thoracoscopic approaches were necessary to remove the tumor without rupturing or damaging the capsule. Therefore, co-operation with the thoracic surgeon was planned.

**Fig. 2:**
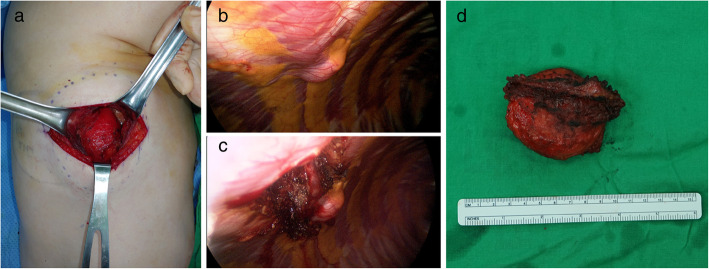
Intraoperative photography. (A) The tumor was exposed under the serratus anterior muscle and separated from surrounding tissue preventing damage to the capsule. (B) Thoracoscopic view showing the herniated tumor in the pleural cavity (C) The en bloc resection of the tumor and intercostal muscle was performed. (D) Excised tumor without damaging the capsule

The patient was laid in the lateral decubitus position. The mass was exposed under the serratus anterior muscle and separated from the surrounding tissue preventing damage to the capsule (Fig. 2a). After full separation until the chest wall, the thoracoscopic approach was started. The tumor was encapsulated by parietal pleura and herniated into the pleural cavity (Fig. 2b). Under combined direct and thoracoscopic views, the tumor was dissected from the rib bone. To avoid capsular injury due to the narrow intercostal space, en bloc resection of the tumor and the intercostal muscle was performed without sacrifying the intercostal vessels and nerve. Finally, the tumor was excised (Fig. 2d).

**Fig. 3:**
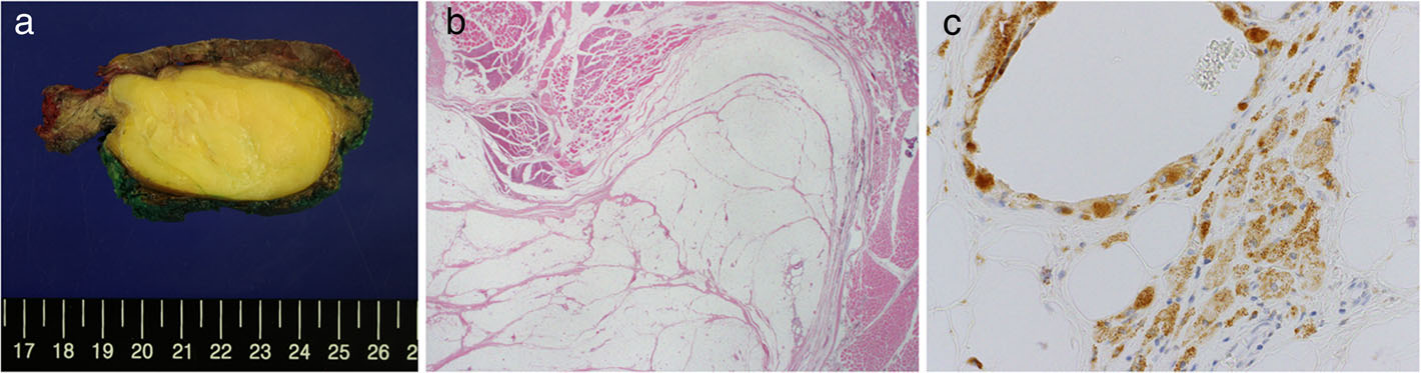
Histological examination consistent with lipoma (a) gross finding (b) Hematoxylin-eosin stain × 12.5 (c) CD68 stain × 400

On the first postoperative day, the chest tube was removed and the patient was discharged on the 4th postoperative day without any specific complications. The pathologic report was consistent with that of a lipoma with focal fat necrosis. For further evaluation of whether the cause of fat necrosis was inflammation or sarcomatous in origin, immunohistochemical staining was performed. CD68, which is a macrophage marker was positive (Fig. 3). The patient did not complain of intercostal neuralgia at the outpatient clinic follow-up.

## Discussion and conclusions

Lipomas have a benign nature and can be treated successfully with surgery. Subcutaneous lipomas can be treated simply; however, during the treatment of atypical deep-seated lipomas, various considerations are needed before surgery. The most representative one is well-differentiated liposarcoma.

The differential diagnosis between atypical deep-seated lipoma and well-differentiated liposarcoma is difficult using imaging modalities such as MR imaging and computed tomography. The accuracy of diagnosis varies among studies from 69 to 83% [7, 8]. In addition to this, there are difficulties in discerning well-differentiated liposarcomas from lipomas associated with sampling error during biopsy [9, 10]. Although common locations of liposarcomas are extremities and the retroperitoneum [11, 12], we should have considered well-differentiated liposarcoma according to MR imaging findings.

The local recurrence of well-differentiated liposarcomas is reported as 10 to 52% [13]. Therefore, resection of the tumor without injury to the capsule is important. We applied both transcutaneous and thoracoscopic approaches, as the best choice for this case, and successfully resected the tumor without injury.

Although the thoracic area is a rare location for lipomas, there have been several reports on it. Endobronchial lipoma [14], diaphragmatic lipoma [15], intramuscular lipoma [5], pleural lipoma [2, 3], and intracardiac lipoma [1, 16] are those that were reported. To our knowledge, a chest wall lipoma growing into the pleural cavity like in this case is extremely rare. A similar case has been reported; however, unlike this case, the portion related to the pleural cavity was large and that of the chest wall was small [6].

Lipomas occasionally present with a dumbbell shape due to the difference in pressures of surrounding structures [17–19]. Cases with such shape need a more complex surgical approach. Even though thoracoscopic surgery is minimally invasive, parietal pleura can be injured through the port site. There is also the risk of developing intercostal neuralgia in thoracoscopic surgery. Therefore, if chest wall lipoma is seated in or near intercostal muscles, early surgical excision should be considered to avoid a complex surgical approach.

## Data Availability

Not applicable.

## Abbreviation

MR: Magnetic resonance
